# The Characteristics of Adjacent Anatomy of Mandibular Third Molar Germs: A CBCT Pilot Study in Patients with Osteogenesis Imperfecta

**DOI:** 10.3390/healthcare8040372

**Published:** 2020-09-30

**Authors:** Giacomo D’Angeli, Daniela Messineo, Mara Riminucci, Alessandro Corsi, Mauro Celli, Iole Vozza, Gian Luca Sfasciotti

**Affiliations:** 1 Department of Oral and Maxillo-Facial Sciences, Sapienza University of Rome, 00161 Rome, Italy; iole.vozza@uniroma1.it (I.V.); gianluca.sfasciotti@uniroma1.it (G.L.S.); 2 Department of Radiological, Oncology and Anatomo-Pathological Sciences, Sapienza University of Rome, 00161 Rome, Italy; 3 Department of Molecular Medicine, Sapienza University of Rome, 00161 Rome, Italy; mara.riminucci@uniroma1.it (M.R.); alessandro.corsi@uniroma1.it (A.C.); 4 Department of Pediatrics and Infantile Neuropsychiatrics, Umberto I Hospital: Rare Bone Metabolism Center, Sapienza University of Rome, 00161 Rome, Italy; mauro.celli@uniroma1.it

**Keywords:** osteogenesis imperfecta, cone beam computed tomography, third molar germs, child, bisphosphonates, pediatric dentistry

## Abstract

(1) Objectives: The aim of our study was to investigate the anatomical features of lower third molar and its adjacent anatomical connections in type I Osteogenesis Imperfecta (OI) patients through cone beam computed tomography (cbct). (2) Methods: The study was conducted among 25 patients, 13 patients with type I OI and 12 control patients (individuals with no disorders and no treatment); average age was 15.44 ± 2.06, 23 third molar germs for each group. The germs have been compared to the parameters using the Mann-Whitney test. A chi-square test was also used to investigate the correlation between the status case/control and tooth development stage. (3) Results: Mann-Whitney test showed significant differences between cases and controls: diameter of the tooth germ in toto (U = 93.5; *p* < 0.001), tooth development stage, (U = 145; *p* < 0.01), roots length (U = 44.5; *p* < 0.01), cementoenamel junction diameter (U = 157.5; *p* < 0.05), size of the pulp chamber (U = 95.5; *p* < 0.05). Type I OI is not associated with the relationship between the germ of mandibular third molar and alveolar canal on axial plane (χ^2^ = 4.095; *p* = 0.129), and parasagittal (χ^2^ = 4.800; *p* = 0.091). The association between type I OI and relationship with the germ of mandibular third molar and alveolar canal on the coronal plane has been significant (χ^2^ = 9.778; *p* < 0.05) as the perforation of the lingual cortical bone in the region of mandibular third molar tooth germ (χ^2^ = 11.189; *p* < 0.01). (4) Conclusions: The results confirm the cbct accuracy in the evaluation of bone density in type I OI patients giving also the opportunity to study the tridimensional anatomy of germs and the adjacent anatomical structures in order to avoid any perioperative complications.

## 1. Introduction

The osteogenesis imperfecta (OI) is a rare hereditary disorder of connective tissue characterized by bone fragility with recurrent multiple fractures from mild trauma and low bone density [1].

Clinical features include short stature, blue sclerae, hearing loss in adulthood, dentinogenesis imperfecta, scoliosis, ligamentous laxity and skeletal deformity.

The OI classification dates back 1979 when Sillence et al. [2] proposed a classification of patients with OI in 4 groups considering clinical criteria and disease severity.

Of OI cases, 90% are caused by mutations in the collagen type I alpha 1 gene (COL1A1) and collagen type I alpha 2 gene (COL1A2), which code for the α1/α2 chains of type 1 collagen, which is the major structural protein of bone [3].

Osteogenesis imperfecta is diagnosed clinically and radiologically [3]. The monitoring exam of OI is the Dual-Energy X-ray Absorptiometry (DXA), which is performed every year to check bone mineral density. If the DXA, after being evaluated by the pediatrician, shows stability bone, pharmacological treatment may be stopped.

Until today, a causal treatment has not been found [4]. The elective treatment is the administration of bisphosphonates [5], a class of drugs classified as bone suppressors, which inhibit osteoclasts and bone remodeling, in order to improve bone resistance to fracture.

Depending on the amine group, bisphosphonates can be divided into two groups: aminoBP and non-amino BP. These drugs are known for the risk of developing osteonecrosis (ONJ) [6,7].

The aim of our study was to investigate the eventual anomalies of lower third molar and its adjacent anatomical connections in type I OI patients comparing the results to a control group (individuals with no disorders and no treatment) through cone beam computed tomography (CBCT).

### Development of the Mandibular Third Molar

The mandibular third molar is the most frequently impacted tooth and represents a surgical because several indications need to be carefully considered for it proper removal. It is very important to understand of the development and movement of the third molar between the ages of 7 and 25 years.

Several longitudinal studies have analyzed the development and eruption of the mandibular third molar [8,9,10,11,12]. Its germ is mostly observable radiographically by the 9th year of age, and the mineralization of the cusp is completed nearly after 2 years. At the 11th year of age, the tooth is placed within the frontal border of the ramus, and its occlusal surface stands nearly anteriorly. The tooth germ’s height is almost occlusal compared to the erupted dentition. Crown formation is usually completed by age14, and the roots are approximately 50% developed by age 16. During this period, the body of the mandible develops in length while the front border of the ramus reabsorbs itself. As this process develops, the third molar places itself near the root of the next second molar. This is accompanied by the development of a more horizontal crown angulation. Usually the roots are totally developed with an open apex by the age 18. By age 24, 95% of all third molars have finished their eruption.

The variation in direction of the occlusal surface from a straight anterior to a straight vertical inclination appears mainly during root growth.

Throughout this period, the tooth rotates from horizontal to mesioangular to vertical, and it achieves its final position by age 20 years. However, approximately half third molars stay as mesioangular impacted teeth [13,14,15].

## 2. Important Factors Which Contribute to Failure of Tooth Eruption

The improper eruption sequence is known as “primary failure of eruption” [16]. When teeth fail to erupt they remain impacted. However, dentoalveolar development continues around these teeth, and a normal dentoalveolar height is reached by periosteal growth formed around the adjacent teeth.

This condition may affect all teeth especially those in the posterior portion. This occurrence can be also noted in type III and type IV OI patients who acquire severe lateral open bites during growth. The dentoalveolar process fails to develop in the area of the non-erupting tooth.

The tooth is usually ankylosed and will not react to orthodontic traction suggesting that the eruptive processes and the typical bone remodeling are absent.

Ankylosis happens when the root cement blends with the dentoalveolar process, and it may be complete or partial when only few parts of the root blend with alveolar process. Where ankylosis occurs, the bone remodeling stops, and the tooth remains impacted.

The insufficient or severely decreased dentoalveolar development is related to this situation.

While the dental crypt goes toward the oral cavity, the bone resorption occurs at the occlusal surface of the crypt, and bone apposition appears at the bottom. Meanwhile the bone formation enlarges the size of the dentoalveolar process.

The complete alveolar bone remodeling happens when permanent teeth substitute deciduous teeth because the alveolar bone associated with the primary tooth is completely reabsorbed together with the tooth roots, and a new alveolar bone is formed [17].

The eruption sequence starts from anterior to posterior sections of the oral cavity. Some factors such as osteoprotegerin, Receptor activator of nuclear factor kappa-Β ligand (RANKL), macrophage colony-stimulating factor (CSF-1) and vascular endothelial growth factor, (VEGF) control the sequence of eruption, but their interactivity is still to be discussed [18,19].

Dental follicles of incisors, canines, and permanent premolars reabsorb the bone and the structure of the primary dental root [20].

Odontoclasts are actively involved in this process [21], and they are characterized by an over expression of RANKL and a reduction of osteoprotegerin (OPG) [22,23].

The odontoclast inactivity causes the non-resorption of primary teeth [24].

Substitute teeth continue to develop, but the lack of the resorption of primary teeth represents an obstacle to eruption leading to an alteration of germs eruption [25]. In patients with type III and IV osteogenesis imperfecta with multiple dental inclusions, an alteration in osteoclast activity was reported in relation to primary teeth resorption [26,27].

## 3. Materials and Methods

This observational study was conducted among 25 patients, 13 patients with OI type I and 12 healthy control patients (CTR).

Inclusion criteria: patients with OI and with lower third molar germs, healthy patients with no disorders and no treatment.

Exclusion criteria: patients without lower third molar germs, patients with osteolitic lesion of the lower third molar germ, second molar missing.

OI was diagnosed clinically and radiologically and molecularly confirmed.

The patients have been primarily visited at Policlinico Umberto I, University Hospital of Rome, Rare Disease Center Skeletal Dysplasia-Bone Metabolic Pathologies, and later at Policlinico Umberto I, University Hospital of Rome, Head and Neck Department, Pediatric Dentistry Unit.

Successively, 46 germs have been considered, 23 of them belonging to OI type 1 patients and the other 23 ones to control patients.

The study protocol complied with the Guidelines for Good Clinical Practice, according to the Declaration of Helsinki (1975). The study was approved by the Institutional Review Board of territorial NHS facilities (n. 260919).

Patients or their legal guardians signed their surgical and radiographical informed consents.

### Instruments and Procedures

**Figure 1:**
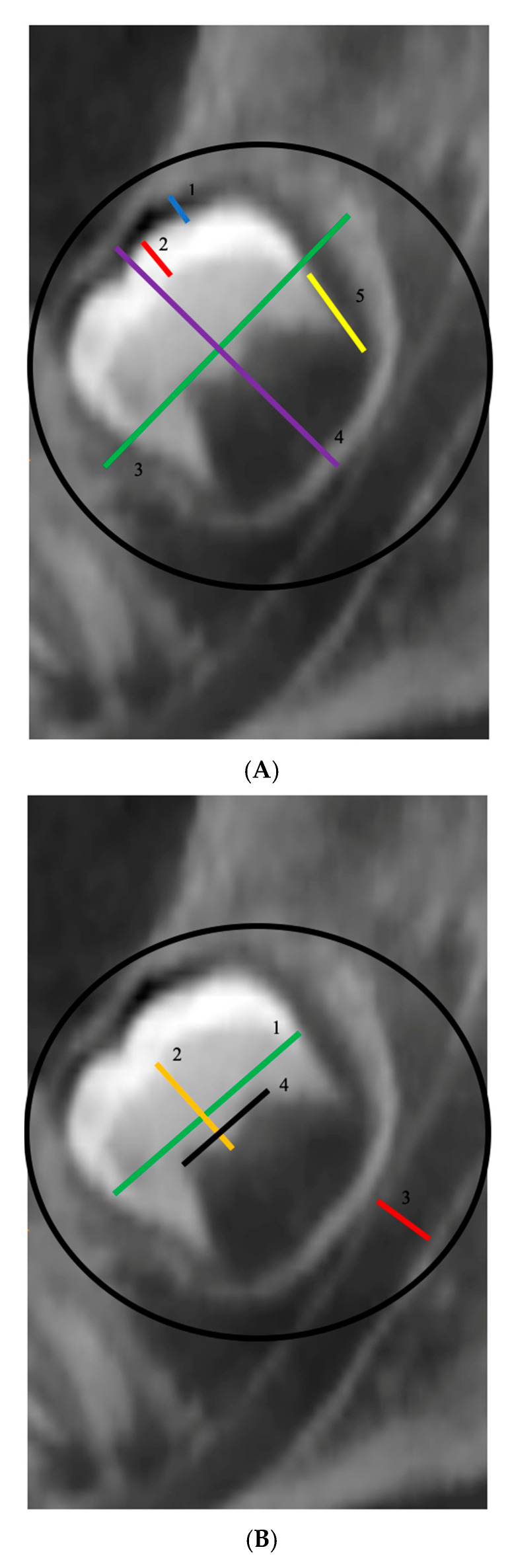
(**A**) Tooth Germ. 1. Width of peri coronal space. 2. Enamel thickness. 3. Diameter of the tooth germ transversal level. 4. Diameter of the tooth germ longitudinal level. 5. Root length. (**B**) Tooth Germ. 1. JEC diameter. 2. Dentin thickness. 3. Alveolar channel diameter. 4. Pulp chamber diameter.

Cone-beam computed tomography has been used to value different parameters on the coronal plane. Imaging was performed with a Newtom 5G device (Cefla S.C., Verona, Italy). Device settings were set at 8 mA and 90 kV. Each field of view (FOV) mode was 5 × 5 cm, and with an isotropic voxel size of 0.4 mm. The width of pericoronal space [28], the enamel thickness, the maximus diameter of the tooth germ from a transversal and longitudinal point of view, root length, (Figure 1A) diameter of the pulp chamber, cementoenamel junction diameter, dentin thickness, the diameter of alveolar canal, (Figure 1B) the stage of tooth formation according to Nolla classification (1961) [29,30], the position of the mandibular third molar germ according to Winter classification (2018) [28].

Regions of interest (ROI) that have been considered are between the second and third mandibular molar, on the dental papilla, after the third molar tooth germ, on the lamina dura, enamel, dentin, pulp chamber and cortical bone.

Another evaluation was based on grayscale level correlating it to bone structure aspect.

## 4. Data Analysis

The software used for data analyses was R. The germ of OI type I patients and the germs of control patients have been compared to the parameters using the Mann-Whitney test.

A chi-square test (Fisher’s exact test) was also used to investigate the correlation between the status case/control and tooth development stage according to Fields classification 95 [31]:

A = initial calcification 8–9 years; B = crown complete at 14 years; C = Eruption 20 years; D = roots complete at 22 years.

Axial, parasagittal, coronal planes are valued according to Ghaeminia and Sun classifications with states I, IIA, IIB and IIC [32,33].

The significance level was set at 0.05.

## 5. Results

This observational study was conducted among 25 patients, 13 patients with OI type I (52%) and 12 healthy control patients (CTR) (48%). The first group was composed by 5 females and 8 males (mean age 16.61) while the second group was made by 5 females and 7 males (mean age 16). The average age was 15.44 ± 2.06 (range 12–19 years old).

### 5.1. Comparing Groups

Mann-Whitney test showed significant difference between the cases and controls: diameter of the tooth germ in toto (U = 93.5; *p* < 0.001), tooth development stage (U = 145; *p* < 0.01), roots length (U = 44.5; *p* < 0.01), cementoenamel junction diameter (U = 157.5; *p* < 0.05), size of the pulp chamber (U = 95.5; *p* < 0.05).

No significant statistical differences were found for alveolar channel (U = 190; *p* = 0.065) which was slightly higher among control group (Mcases = 2.48 ± 0.66; Mcontrols = 2.83 ± 0.58).

**Figure 2:**
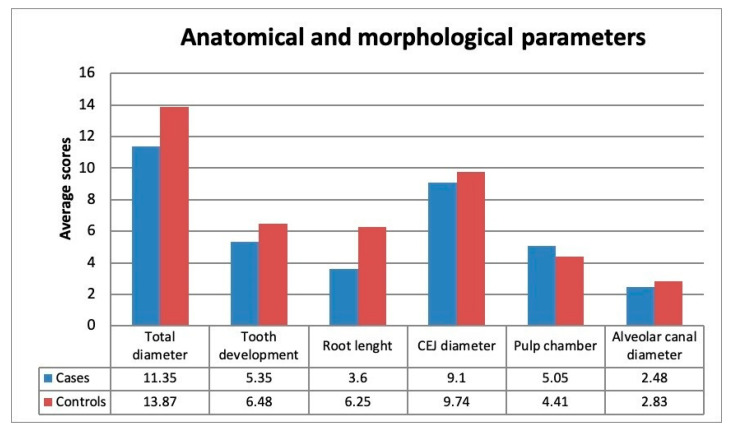
Anatomical and morphological parameters. Data are from the lower third molar germ and adjacent anatomical structures.

Compared to controls, the Average Score Analysis has shown that the germs of patients with type I Osteogenesis imperfecta are characterized by reduction in diameter (Mcases = 11.35 ± 2.04; Mcontrols = 13.87 ± 1.98), lower level of tooth development (Mcases = 5.35 ± 1.33; Mcontrols = 6.48 ± 1.08), lower root length (Mcases = 3.60 ± 2.82; Mcontrols = 6.25 ± 2.24), smaller cementoenamel junction diameter (Mcases = 9.10 ± 1.04; Mcontrols = 9.74 ± 0.86) and larger size of pulp chamber (Mcases = 5.05 ± 0.85; Mcontrols = 4.41 ± 0.71) (Figure 2).

With regard to ROI, significance differences were found between cases and controls about the values between second and third molar (U = 115.5; *p* < 0.01), dental papilla (U = 164; *p* < 0.05), after the germ of mandibular third molar (U = 169; *p* < 0.05) and enamel (U = 163; *p* < 0.05).

No differences were found between the cases and controls with reference to the values of lamina dura (U = 218; *p* = 0.307), dentin (U = 209; *p* = 0.223), pulp chamber (U = 236; *p* = 0.531) and cortical bone (U = 195; *p* = 0.127).

**Figure 3:**
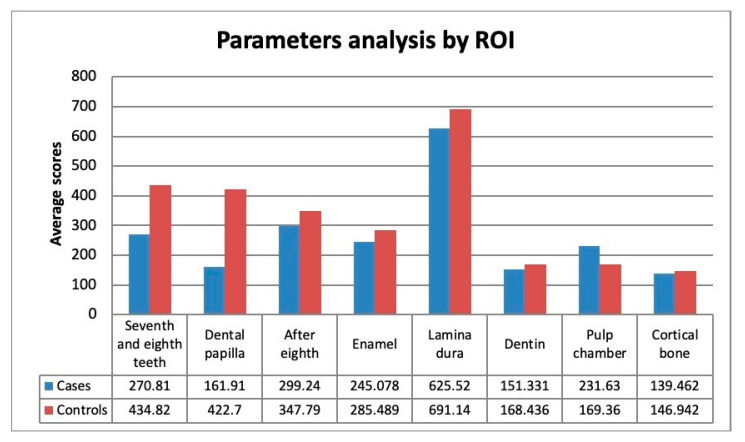
Parameters analysis by ROI. Scores of enamels, lamina dura, dentin and cortical bone are divided by 10 to preserve graph proportions.

Compared to controls, the average score analysis about the values taken from ROI has shown that patients with type I osteogenesis imperfecta have minor density between the second and third molar (Mcases = 270.81 ± 206.96; Mcontrols = 434.82 ± 226.180), minor density at the level of papilla (Mcases = 161.91 ± 330.882; Mcontrols = 422.70 ± 342.487), minor density after the germ of the third molar (Mcases = 299.24 ± 263.798; Mcontrols = 347.79 ± 152.980), minor enamel density (Mcases = 2450.78 ± 681.303; Mcontrols = 2854.89 ± 466.773) (Figure 3).

### 5.2. Association

Type I Osteogenesis imperfecta is not associated with the relationship between the germ of mandibular third molar and alveolar canal on axial plane (χ^2^ = 4,095; *p* = 0.129) and parasagittal (χ^2^ = 4.800; *p* = 0.091). This means, regardless of the pathology, the distribution of subjects in different categories did not change—the ratio germ/inferior alveolar nerve has remained unchanged in both groups.

For example, considering the axial plane of the 23 cases, 22 cases have been classified as I and 1 case as IIA, while among the 23 controls, 20 of them have been classified in I and 3 in IIB.

Considering the parasagittal plane of the 23 cases, 19 have been classified in I, 3 in IIA and 1 case in IIB, while among the 23 controls, 19 have been classified in I and 4 in IIB.

The association between type I Osteogenesis imperfecta and relationship with the germ of mandibular third molar and alveolar canal on the coronal plane has been significant (χ^2^ = 9.778; *p* < 0.05).

Among the 23 cases, 22 have been classified in I and 1 case in IIA, while among the 23 controls, 14 have been classified in I, 1 one case in IIA, 3 in IIB and 5 in IIC. Therefore, more controls have been categorized into IIB and IIC categories than the cases.

No relevant association was detected between type I Osteogenesis Imperfecta and tooth development stage according to Proffit e Fields 1995 classification (χ^2^ = 2.091; *p* = 0.489) and dentin alteration according to Shields et al. 1973 classification (χ^2^ = 3.748; *p* = 0.091).

Lastly a significant association has been found between type I Osteogenesis Imperfecta and the perforation of the lingual cortical bone in the region of mandibular third molar tooth germ (χ^2^ = 11.189; *p* < 0.01). In particular, unlike the control group, 9 out of 23 cases, has shown perforation of the lingual cortical bone (Table 1).

## 6. Discussion

Over the last decade, CBCT has been recognized as a transverse cross-section potentially low dosage to visualize bone structures on the head and neck region and it became one of the most common medical imaging techniques.

For Oral Maxillo Facial exams (OMF), you can predict an effective dose in the interval 30–80 microSV in the most scans depending on FOV e cbct parameters [34,35,36,37,38].

As said before and as found in literature, regarding the smaller size of the tooth germ in toto, smaller size of the roots, low level of mandibular third molar development, enamel density, size of the pulp chamber in OI patients, we can say that, compared to the control group, there is a growth defect due to disease.

Although tridimensional radiology technique as cbct had never been used before, the literature confirms our data. Levin LS et al. (1981) agreed on smaller germ diameter in toto, smaller cementoenamel junction diameter, low level of tooth development [26,39,40,41,42].

Radiology confirms minor root length, larger size of pulp chamber and low-density enamel.

Teeth elements have the typical ghost aspect:

Large pulp chambers, large root canals due to hypoplasia of the dentin which outlines a very short root. The enamel is thin, less thick and poorly mineralized and it may not be evident on two-dimensional radiography.

The tooth appears to be like a thin shell and those which do not erupt, reabsorb [43].

Regarding to the relationship with the inferior alveolar nerve (IAN) and the presence of germs with disrupted lingual cortex we can assume a delayed growth of the mandibular third molar as Panzoni E. e Covani U. described. They found how the mandibular third molar tooth germ originate lingually and then it goes buccally to the second molar and behind it [44,45].

With reference to dentin density we can state that no alterations regarding DI, compared to controls, have been found. This has been confirmed by clinical cases.

No OI patients had DI [46,47].

To confirm low bone density detected between second and third molar and after third molar in our study, Parfitt et al. conducted a study enrolling 70 children with type I, type III and IV OI aged between 1.5–13.5 in which cortical width was generally lower than normal.

Since the bone size and cortical width during growth are defined by the remodeling process, OI patients are subject to a bone remodeling defect. This is an essential aspect of the pathology because a poor bone remodeling will lead to lesser cross-sections and thinner bone cortex in long bones, thus reducing overall bone strength.

In addition to the aforementioned defects of the bone, OI is also characterized by a lesser number of bone trabeculae leading to a more cancellous bone structure. An increase loss or decrease in production of trabeculae may be related to a low trabecular number. There was no evidence that children affected by OI lose secondary trabeculae since the trabecular number remained steady with increasing age, therefore suggesting fewer secondary trabeculae produced.

Bone trabeculae consist of lamellar bone of which the composing lamellae appear to be thinner in affected children as opposed to healthy counterparts. Inadequate lamellar thickening in OI appears to be linked to the bone remodeling defect; in the control group, each remodeling cycle added 2.8 µm of bone thickness.

Amongst the different types of OI, type I OI showed a positive bone balance of 1.1 µm, whilst type III and IV showed approximately 0 µm of bone thickness gain. Since the production carried out by a single cell was decreased by 50%, the performance of the osteoblasts was insufficient. This was only partly compensated by an increase in the number of osteoclasts per remodeling unit.

Although the amount of the transformed bone in individual remodeling cycles is decreased in OI, the number of remodeling cycles that occur on a certain bone surface per unit time is increased [48].

Conducted by Nagai N et al., Thomson CA et al., Fedarko NS et al., the in vitro studies of osteoblasts taken from OI humans and Osteogenesis Imperfecta Murine (OIM) mice showed a decrease in markers of osteoblast differentiation along with a reduced rate in cell proliferation. If this characteristic of OI extends to in vivo scenarios, it may be a secondary factor of the bone disease since not only is there an impairment in the quantity or quality of the matrix formed but even a decreased number of differentiated osteoblasts producing mineralized bone matrix. There is a link between type I collagen synthesis and cell production suggesting growth impairment in OI type I patients.

The mechanism of low level of cell proliferation and differentiation of osteoblasts could be a direct consequence of the retained procollagen molecules with the distended rough endoplasmatic reticulum, which may indirectly have an effect on the quality and quantity of the extracellular matrix produced by the proteoblastic cell necessary for osteoblastic differentiation [49,50,51].

The high rate of bone turnover of the disease may lead to exhaustion and/or premature senescence of stem cells capable of generating osteoclastic cells in vitro, which if present in intact bone, will further contribute to the severity of the bone disease, particularly in OI elderly patients [52,53].

Alanay Y. et al. [54] and Rauch F et al. [55] found that intact bone is able to perceive when a defective matrix develops, usually due to microfractures, and start a remodeling cycle to renew the bone.

This essential principle of bone physiology is constantly present in OI patients, given that the weaker bone matrix is continuously subject to microfractures. Histologically speaking, the OI bone reveals a state of unproductive bone formation and an increase in the numbers of osteoclasts and osteocytes.

In type I OI, the amount of bone formed during a remodeling cycle is decreased compared to controls.

However, we have to underline a limitation of our study linked to a limited sample size: Further studies with an increased sample size are necessary in order to confirm our results.

## 7. Conclusions

The results from our study confirm the existence of anomalies of lower third molar and its adjacent anatomical connections in type I OI patients comparing the results to a control healthy group through cbct. The cbct accuracy in the evaluation of bone density in type I OI patients and healthy patients shows particular radiographic features of teeth elements in OI patients and low bone density due to the disease.

Moreover, our results indicate that volumetric measurements by cbct (volume cbct) are a not invasive and a reliable method to value the condition of third molar germs for the purposes of the a future surgical extraction. We have also the opportunity to study the tridimensional anatomy of the germ itself and the adjacent anatomical structures in order to avoid any perioperative complications.

Considering these patients are eligible for a mandibular third molar germectomy, further studies will focus on biopsy of bone sample from alveolar socket in order to investigate the alterations as described in the literature and compare them to alveolar socket healing.

## Figures and Tables

**Table 1:** Results associations between OI and healthy patients.

	Axial			
	I	IIA	IIB	IIC
Cases	22 (95.6%)	1 (4.4%)	0	0
Controls	20 (87%)	0	3 (13%)	0
	**Parasagittal**			
	I	IIA	IIB	IIC
Cases	19 (82.6%)	3 (13%)	1 (4.4%)	0
Controls	19 (82.6%)	0	4 (17.4%)	0
	**Coronal**			
	I	IIA	IIB	IIC
Cases	22 (95.6%)	1 (4.4%)	0	0
Controls	14 (60.9%)	1 (4.4%)	3 (13%)	5 (21.7%)
	**State of Growth**			
	A		B	
Cases	2 (8.7%)		21 (91.3%)	
Controls	0		23 (100%)	
	**Altered State of Dentin**			
	No		Yes	
Cases	16 (84.2%)		3 (15.8%)	
Controls	22 (100%)		0	
	**Interruption of the Lingual Cortical Plate on the Axial Axis Cone-Beam**			
	No		Yes	
Cases	14 (60.8%)		9 (39.2%)	
Controls	23 (100%)		0	

Altered state of dentin in 4 OI patients and 1 healthy patient could not be evaluated.
